# Historical versus Contemporary Climate Forcing on the Annual Nesting Variability of Loggerhead Sea Turtles in the Northwest Atlantic Ocean

**DOI:** 10.1371/journal.pone.0081097

**Published:** 2013-12-05

**Authors:** Michael D. Arendt, Jeffrey A. Schwenter, Blair E. Witherington, Anne B. Meylan, Vincent S. Saba

**Affiliations:** 1 Marine Resources Division, South Carolina Department of Natural Resources, Charleston, South Carolina, United States of America; 2 Department of Biological Sciences, University of South Carolina, Columbia, South Carolina, United States of America; 3 Fish and Wildlife Research Institute, Florida Fish and Wildlife Conservation Commission, Melbourne Beach, Florida, United States of America; 4 Fish and Wildlife Research Institute, Florida Fish and Wildlife Conservation Commission, St. Petersburg, Florida, United States of America; 5 National Marine Fisheries Service, National Oceanic and Atmospheric Administration, Princeton, New Jersey, United States of America; Monash University, Australia

## Abstract

A recent analysis suggested that historical climate forcing on the oceanic habitat of neonate sea turtles explained two-thirds of interannual variability in contemporary loggerhead (*Caretta caretta*) sea turtle nest counts in Florida, where nearly 90% of all nesting by this species in the Northwest Atlantic Ocean occurs. Here, we show that associations between annual nest counts and climate conditions decades prior to nest counts and those conditions one year prior to nest counts were not significantly different. Examination of annual nest count and climate data revealed that statistical artifacts influenced the reported 31-year lag association with nest counts. The projected importance of age 31 neophytes to annual nest counts between 2020 and 2043 was modeled using observed nest counts between 1989 and 2012. Assuming consistent survival rates among cohorts for a 5% population growth trajectory and that one third of the mature female population nests annually, the 41% decline in annual nest counts observed during 1998–2007 was not projected for 2029–2038. This finding suggests that annual nest count trends are more influenced by remigrants than neophytes. Projections under the 5% population growth scenario also suggest that the Peninsular Recovery Unit could attain the demographic recovery criteria of 106,100 annual nests by 2027 if nest counts in 2019 are at least comparable to 2012. Because the first year of life represents only 4% of the time elapsed through age 31, cumulative survival at sea across decades explains most cohort variability, and thus, remigrant population size. Pursuant to the U.S. Endangered Species Act, staggered implementation of protection measures for all loggerhead life stages has taken place since the 1970s. We suggest that the 1998–2007 nesting decline represented a lagged perturbation response to historical anthropogenic impacts, and that subsequent nest count increases since 2008 reflect a potential recovery response.

## Introduction

Across numerous disciplines, data are collected for the purpose of monitoring change and projecting trends. The value of these projections is contingent upon the quality of both the input data and the analytical model. Because inaccuracies in short-term model fits are compounded over time, the accuracy of long-term projections reflects the reliability of short-term predictors [1]. As such, the number of temporal iterations upon which a projection is based is also of paramount importance. For demographic research, this caveat implies that population projections for long-lived, late maturing species may be less robust than for short-lived, early maturing species simply due to the number of years required to reach maturity.

Sea turtles are long-lived, late maturing species that are globally distributed but not adequately globally protected [2]. Conant et al. [3] modeled life history parameters and annual survival rates for nine populations of loggerhead sea turtles (*Caretta caretta*) under the assumption of population growth, and concluded that this species was especially vulnerable to perturbations. Extrapolation of these life history parameters and annual survival rates for loggerhead sea turtles in the Northwest (NW) Atlantic Ocean suggests that only 0.2% of a given cohort remains by age 30 assuming a 5% (λ = 1.05) population growth rate. Because survivorship and reproductive values are highest for sea turtles that have reached or are approaching maturity [4], [5], protecting these life stages from anthropogenic threats such as commercial fishing is especially important [6].

Van Houtan and Halley [7] recently proposed an alternative to the long-held paradigm that the survivorship of large juvenile and adult sea turtles is more predictive of population change than juvenile recruitment. Although they did not exonerate fisheries mortality as an important threat, Van Houtan and Halley [7] suggested that cohort effects stemming from survival in the first year of life had a greater effect on population growth, concluding that this factor explained more than two-thirds of future interannual variability in loggerhead sea turtle nest counts in Florida. Their premise was that environmental conditions, based on examination of a 31-year lag in the Atlantic Multi-Decadal Oscillation (AMO), drive circulation patterns that in turn affect food availability and hatchling dispersal, and thus, hatchling survival [7]. Because 80–90% of all nesting by this species in the NW Atlantic occurs in Florida [8], this conclusion holds substantial implications for subsequent management of this population [3].

The mechanism proposed by Van Houtan and Halley [7] was based on well-documented environmental influences on larval fish dispersal and survival [9]; however, hatchling sea turtles may be better equipped than larval fish to survive harsh conditions between ages 0 and 1 year. Many fish species have r-selected traits in that they mature early, have a high fecundity, and are short-lived, whereas all sea turtle species have mostly k-selected traits which include late maturity, lower fecundity (at least relative to fishes), and long lifetimes [10]. Therefore, survival of young-of-the-year would affect adult recruitment states in fishes more so than in sea turtles, simply due to earlier maturity in most fishes. Although neonate survival within the nest environment (i.e., hatching success, emergence rate, poaching, predation, etc.) is likely to affect adult recruitment in sea turtles [11], [12], sea turtle adult recruitment is less sensitive to variable survival at the in-water neonate stage relative to fishes. Furthermore, because loggerhead sea turtle hatchlings are “low-energy float and wait foragers” [13], sea turtle hatchlings should be able to better withstand longer bouts of reduced food availability than larval fish.

In contrast to hatchlings, adult female sea turtles may be greatly influenced by environmental conditions, at least in regards to annual reproductive activity [14], [15], [16], [17], [18], [19]. Not all adult female sea turtles in a population nest each year, which Hays [20] attributed to rates of energy consumption on the foraging grounds in the years prior to nesting. Accordingly, the 31-year lag effect reported by Van Houtan and Halley [7] may not actually capture age 0 survival, but rather, it may reflect the influence of winter and spring climate conditions on adult female energy uptake and thus, the number of annual nests laid. If true, contemporary environmental conditions may exert greater influence on annual nest counts than suggested by Van Houtan and Halley [7]. With this hypothesis in mind, the first objective of the present study was to statistically evaluate the relative influence of the AMO, as well as two other important and inter-related climate indices in the northern hemisphere (i.e., the North Atlantic Oscillation (NAO) and the El Niño Southern Oscillation (ENSO) [21]), at both contemporary and historical intervals using the models considered by Van Houtan and Halley [7]. A second objective was to test for differences in model fits using temporally partitioned annual nest count data to assess variability in model performance with respect to environmental metrics. A third objective was to assess the relative contribution of first time nesters (neophytes) to projected annual nest counts through the year 2043, which was suggested by Van Houtan and Halley [7] to be a mechanism for generational replication of cohort effects. Lastly, a survival matrix was created in order to quantitatively compare the relative importance of survival in the first year of life relative to the next 30 years before reaching maturity.

## Methods

### Data Selection and Manipulation

Annual loggerhead sea turtle nest count data at the 15 Florida index beaches analyzed by Van Houtan and Halley [7] were obtained directly from the Florida Fish and Wildlife Conservation Commission. All 15 beaches were located on the Atlantic coast of Florida, U S A between 28.7°N (Canaveral National Seashore) and 26.5°N (Boca Raton). Between 1989 and 2012, the period of record for the present study, these 15 beaches comprised an average of 67.6% of total annual nest counts reported from all beaches in Florida via the Statewide Nesting Beach Survey program (ABM, pers. obs). Because annual nest count data between 1989 and 2012 were normally distributed, actual values, rather than ln-transformed values [7], were analyzed. Given similar temporal trends across beaches [8], pooled annual nest count data were analyzed.

Unsmoothed (standard) monthly AMO data between 1856 and 2012 (long format) were uploaded from the Earth Systems Research Laboratory (ESRL) of the National Oceanographic and Atmospheric Administration (NOAA); http://www.esrl.noaa.gov/psd/data/timeseries/AMO/. Because the AMO index is based on sea surface temperature anomalies, reported values are dynamic rather than static. This practice results in high temporal variability for reported values; however, correspondence between data set versions also remains high. For example, annualized (by calendar year) AMO values analyzed in the present study differed from data analyzed by Van Houtan and Halley [7] by 1.9±35.6% (mean ± standard deviation [SD]); however, the Pearson’s correlation co-efficient (r) between the normalized versions of these two data sets (1856–2010) was 1.0. The AMO data were normalized as in Van Houtan and Halley [7] and confirmed through personal communication (K. Van Houtan, email, 2 January 2013): a single annual value was created as the mean of monthly values between January and June plus the preceding December, from which the grand mean was subtracted and the resulting value divided by the grand SD, in order to generate a normalized series with a mean of 0 and a SD of 1.

Gridded (1°×1°) monthly mean sea surface temperature (SST) data from the Optimal Interpolation (OI) version 2 satellite were obtained from the ESRL for the same spatial range (22–38°N, 72–84°W; http://www.esrl.noaa.gov/psd/data/gridded/data.noaa.oisst.v2.html ) specified by Van Houtan and Halley [7]. This temporal range covered the majority of the geographic distribution of dispersing hatchlings, neritic juveniles, and adult loggerheads from the stock nesting principally on Florida beaches. We reproduced analyses of Van Houtan and Halley [7] by including only data for December in the year prior to nesting for grid cells identified by the ESRL as not containing land coverage (once data were averaged to a single yearly value).

Monthly values for the NAO and running tri-monthly values for the ENSO between 1950 and 2012 were uploaded from the Climate Prediction Center of the NOAA National Weather Service (http://www.cpc.ncep.noaa.gov/products/precip/CWlink/pna/nao.shtml; http://www.cpc.ncep.noaa.gov/products/analysis_monitoring/ensostuff/ensoyears.shtml). Despite both of these data sets being indices, values remained static over time; thus, unlike the AMO, original data, rather than normalized data, were used for analyses.

### Analyses

For objective 1, a series of hierarchical cluster analyses (Euclidean distance) were performed in Minitab 15® (Minitab, Inc., State College, Pennsylvania, U S A) to evaluate changes in fit between annual loggerhead sea turtle nest counts and AMO, NAO, and ENSO in the same year of nesting and lagged at annual intervals up to 39 years. Analysis of Variance (ANOVA; Minitab 15®) was used to test for differences in the distribution of percent similarity values among these three environmental indices. For all statistical tests, significance level (α) was 0.05. For each index, optimal lag intervals were selected based on maximum percent similarity at both a contemporary scale (<4 years) to encompass remigration rates [3] and at >10 years prior to nest count year to evaluate cohort effects. The later criteria was selected given few reports of age at maturity for loggerhead sea turtles in the NW Atlantic at <10 years [22].

Optimal contemporary and historical lag period influences on annual loggerhead sea turtle nest counts were evaluated using the four general linear models (Minitab 15®) of Van Houtan and Halley [7]: Model 1 = linear fit, single parameter; Model 2 = curvilinear fit, single parameter; Model 3 = model 1+SST; Model 4 = model 2+SST. Parameter entry order was also evaluated as part of the process for selecting a best contemporary lag and best historical lag parameter for subsequent model testing. A paired t-test (Minitab 15®) was used to compare predicted and actual nest counts (1989–2012) based on the best contemporary and historical lag parameters.

General linear models (Minitab 15®) were also used to evaluate the relative influence of the optimal contemporary lag parameter, optimal historical lag parameter, and both parameters combined. These evaluations were conducted for the full 24-year data set, as well as with the data set portioned into two groups denoting the first and last 12 years to compare model fits before and during a precipitous nest count decline [8]. Parameter fits were evaluated using significance (*P*-value) and model fits were described by model-adjusted coefficient of determination (r^2^); because Van Houtan and Halley (2011) reported the correlation coefficient (r) for actual vs. predicted nest counts in lieu of r^2^, we also provide this metric in our tables.

For objective 2, annual nest count data were filtered as two, three, and four year series to evaluate the role of these remigration intervals on interannual variability in nest counts, and in turn, model performance. For each remigration interval, nest counts recorded in 1989 were assigned to the first data series, with the second data series beginning in 1990 and so forth. In order to further evaluate whether optimal contemporary and historical lag parameters were consistently significant throughout the data set, annual nest count data for each series for each remigration interval were described using the best fit equation selected in objective 1.

For objective 3, we applied the life history parameters and annual survival rates reported by Conant et al. [3] for a 5% (λ = 1.05) population growth rate to annual nest counts between 1989 and 2012, in order to calculate the age 31 neophyte nesting population during 2020–2043. The neophyte nesting population at age 31 was computed as follows: nest count in the year of cohort origin ×57.5 female eggs per nest ×54% hatchling emergence ×70% hatchling frenzy survival (initial dispersal) ×40% age 0 survival ×79.4% annual survival during the next 10 years ×89.3% annual survival during the next 19 years ×95% survival during the 31st year, with continued 95% annual survival thereafter. Conant et al. [3] did not incorporate survival for the hatchling frenzy [23]; however, we conservatively estimated it as 70% for this brief but important gauntlet based loosely on published observations [24]. We acknowledge that inclusion of this additional mortality gauntlet reduces cohort first year survival by seven percent and in turn alters modeled population growth rates [3] which are beyond the scope of this study. Also, because the purpose of this analysis was to focus exclusively on changes in future abundance under the assumption of consistency among developing cohorts, we disregarded potential interannual differences in cohort sex ratios [25] and assumed a 1∶1 female to male ratio [3].

In order to evaluate the relative importance of the future neophyte population, we also quantified the size of the remigrant population using the metrics of Conant et al. [3]. Assuming a clutch frequency of five nests per season, we estimated that 11,367 mature female loggerheads nested in 2012. Given a remigration interval of three-years, we multiplied the estimated number of female nesters in 2012 by three to generate a total remigrant female loggerhead population of 34,102 individuals in 2012. Because of the highly variable interannual trends in loggerhead sea turtle nest counts in the past decade, we selected three different scenarios for calculating the remigrant population at the start of the 2020 nesting season: (A) unchanged from 2012, (B) following a 20% annual increase between 2013 and 2019 consistent with interannual trends between 2007 and 2012, and (C) following a 20% annual decrease between 2013 and 2019 for further context. The relative composition of neophytes in 2020 was computed as the number of neophytes surviving to age 31 divided by the sum of these neophytes plus one-third of the total remigrant population alive at the start of the 2020 nesting season, which was 5% less than in 2019. In subsequent years, neophyte nesters from the previous year were included in the remigrant population size calculation after applying the 95% annual survival rate. Reproductive longevity for this species is not well documented, but limited tag-recapture data suggest it may span >25 years [26], which is longer than the duration of the annual nest count data set considered herein.

For objective 4, a survival matrix was created to compare the relative effects of survival in the first year of life (3 stages) vs. the next 30 years using 12 mean survival rates: 1%, 10%, 20%, 30%, 40%, 50%, 60%, 70%, 80%, 90%, 95%, and 99%. Cell values in this matrix denoted the proportion (to 0.00%) of the cohort that remained after 31 years. Maximum annual nest count discrepancies in the 24-year data set for the 15 Florida beaches were extrapolated to evaluate the maximum net increase in annual survival needed to temper cohort size disparity by age 31.

## Results

### Objective 1: Contemporary vs. Historical Model Fits

**Figure 1:**
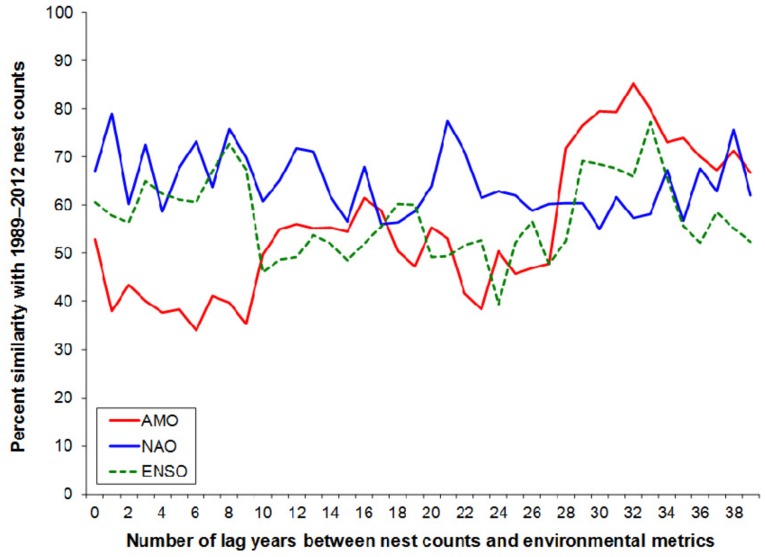
Associations between climate indices and loggerhead (*Caretta caretta*) sea turtle nesting on Florida index beaches. Percent similarity (y-axis) between the NAO (blue line) and annual nest counts at 15 Florida index beaches (1989 to 2012) peaked a year prior to nesting; however, percent similarity for the AMO (red line) and the ENSO (green line) and annual nest counts peaked at 32 and 33 years prior to nesting, respectively.

The distribution of percent similarity values between annual loggerhead sea turtle nest counts (1989–2012) and environmental indices in the same year or lagged annually by up to 39 years were significantly different (F_2_ = 8.31, *P*<0.001) among environmental indices (Figure 1). The NAO was associated with a higher overall percent similarity (mean ±SD = 64±7%) than either the AMO (56±15%) or ENSO (57±8%). Optimal contemporary and historical lag associations for the NAO occurred in March of the year prior to nesting (79% similarity) and in September 20 years prior to nesting (77% similarity), respectively. Lag associations for the AMO and the ENSO always occurred in the last step of each cluster analysis; thus, the original normalized value (AMO) or twelve month annualized (ENSO) values were used. Optimal contemporary AMO (53% similarity) and ENSO (65% similarity) lags occurred the year of nesting and three years prior to nesting, respectively. Optimal historical AMO (85% similarity) and ENSO (77% similarity) lags occurred 32 years and 33 years prior to nesting, respectively.

At contemporary time-scales, the NAO in March of the year prior to the year of nesting was the only significant (*P* = 0.003) parameter in model 1, where it accounted for 34% of the sums of squares (Table S1). The SST term was only significant in models 3 and 4 when the NAO was excluded; thus, the NAO in March of the year prior to nesting was substituted for SST as the contemporary model parameter of choice.

At historical lag scales, the normalized AMO 32 years prior to the year of nesting was the only significant (*P*<0.001) parameter in model 1, where it accounted for 44% of the sums of squares (Table S1). The AMO was a non-significant (*P*>0.05) term in models 2 and 4, but accounted for a high (and variable depending on entry order) proportion of the sums of squares (Table S1). Because the square of negative and positive values disproportionately relate to their original values, which greatly affected the AMO residuals (), we only analyzed the AMO using the linear model instead of AMO+AMO^2^ as in the curvilinear.

**Figure 2:**
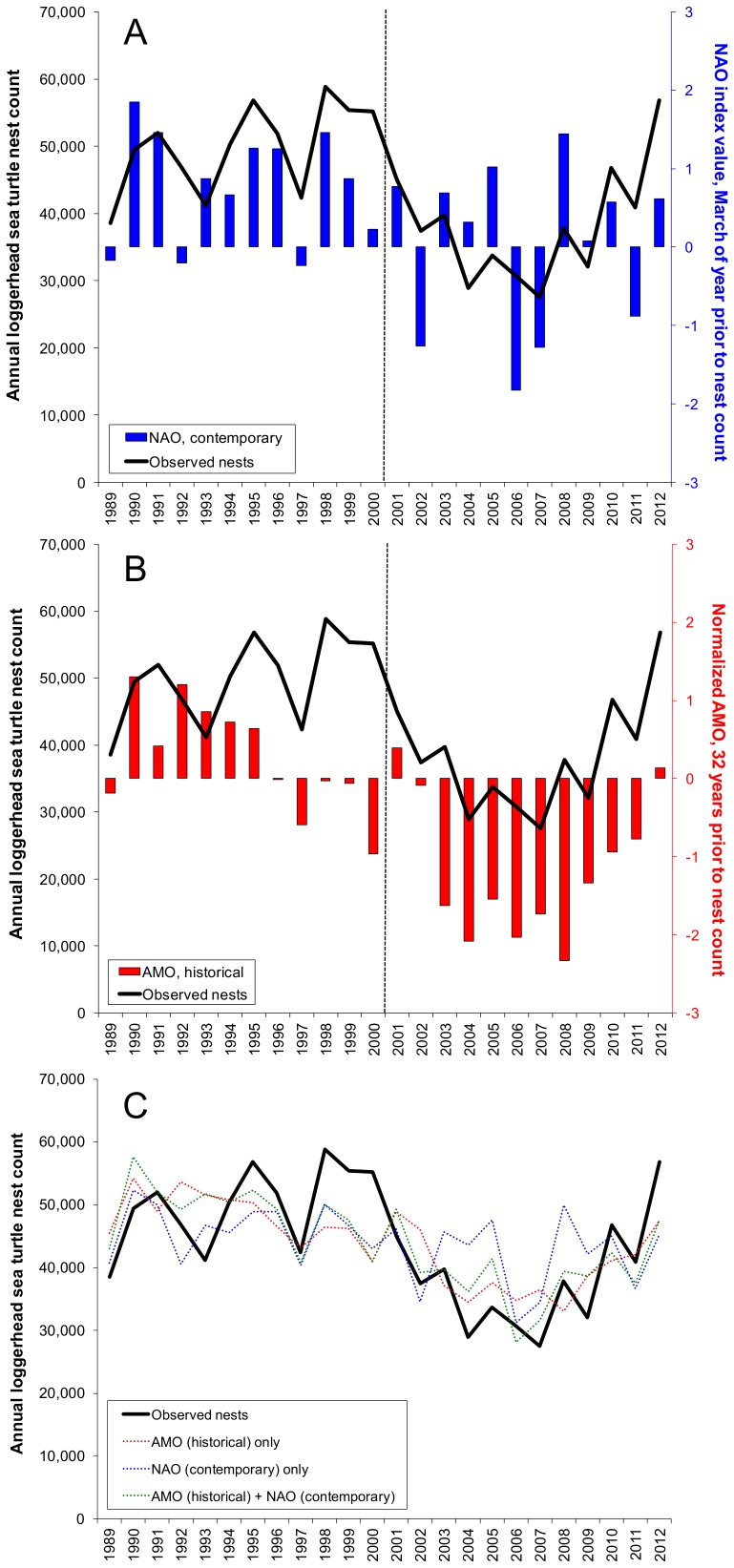
Fitted (A,B) and predicted (C) loggerhead (*Caretta caretta*) sea turtle nests on Florida index beaches. Annual nest counts predicted by contemporary NAO only (blue line), historical AMO only (red line), or both parameters combined (green line) were not statistically different. In all panels, observed annual nest counts for loggerhead (*Caretta caretta*) sea turtle at 15 Florida index beaches during 1989–2012 are displayed as black lines.

Contemporary NAO was a significant model term overall (*P* = 0.006), but not when the data set was partitioned into 12-year spans (Table 1); however, model fits for contemporary NAO were similar during both periods (Figure 2a). Historical AMO was a significant (*P*<0.001) model term overall and after 2001 (*P* = 0.009), but was non-significant (*P* = 0.744) during 1989–2000 (Figure 2b). Annual nest counts predicted by the contemporary NAO only vs. the historical AMO only during 1989–2012 were not significantly different (T = 0.0, P = 1.000; Figure 2c). The greatest co-efficient of determination (adjusted r^2^ = 0.53) and thus the best model fit was observed when both the contemporary NAO and the historical AMO were included (Figure 2c).

**Table 1:** Descriptive statistics for predictions of loggerhead sea turtle (*Caretta caretta*) nest counts at 15 Florida index beaches during 1989 to 2012 based on historical (AMO) and contemporary (NAO) environmental influences.

			AMO		NAO	
Overall data set	adj r^2^	r	P-value	% SS	P-value	%SS
y = 41737+5714*NAO_contemporary_	0.31	0.58	n/a	n/a	0.006*	30
y = 45910+5147*NAO_contemporary (1989–2000)_	0.26	0.57	n/a	n/a	0.053	33
y = 38056+3240*NAO_contemporary (2001–2012)_	0.08	0.40	n/a	n/a	0.192	16
y = 46608+5828*AMO_cohort_	0.41	0.66	<0.001*	56	n/a	n/a
y = 50173 − 983* AMO_cohort (1989–2000)_	0.00	0.11	0.744	1	n/a	n/a
y = 45862+6645* AMO_cohort (2001–2012)_	0.46	0.71	0.009	51	n/a	n/a
y = 44513+4583*AMO_cohort_ +3868*NAO_contemporary_	0.53	0.76	0.003*	23,44	0.018*	13, 34
**2-year remigration interval**	**adj r^2^**	**r**	**P-value**	**% SS**	**P-value**	**%SS**
(1989 series): y = 45495+6976*AMO_cohort_ +3017*NAO_contemporary_	0.62	0.83	0.004*	52, 56	0.082	13, 17
(1990 series): y = 46376+3374*AMO_cohort_ +3551*NAO_contemporary_	0.44	0.74	0.132	14, 49	0.119	15, 40
**3-year remigration interval**	**adj r^2^**	**r**	**P-value**	**% SS**	**P-value**	**%SS**
(1989 series): y = 45495+6976*AMO_cohort_ +3017*NAO_contemporary_	0.60	0.85	0.077	28, 55	0.145	17, 44
(1990 series): y = 43236+2044*AMO_cohort_ +2919*NAO_contemporary_	0.25	0.68	0.447	7, 30	0.273	16, 39
(1991 series): y = 50315+8261*AMO_cohort_ +4749*NAO_contemporary_	0.72	0.90	0.010*	58, 65	0.063	15, 22
**4-year remigration interval**	**adj r^2^**	**r**	**P-value**	**% SS**	**P-value**	**%SS**
(1989 series): y = 40716+4243*AMO_cohort_ +320*NAO_contemporary_	0.42	0.81	0.107	60, 65	0.870	<1, 5
(1990 series): y = 45584+4694*AMO_cohort_ +2300*NAO_contemporary_	0.18	0.71	0.276	29, 41	0.490	10, 22
(1991 series): y = 49103+10090*AMO_cohort_ +3279*NAO_contemporary_	0.87	0.96	0.011*	82, 84	0.168	8, 11
(1992 series): y = 45387+798*AMO_cohort_ +7595*NAO_contemporary_	0.57	0.86	0.824	<1, 43	0.155	31, 73

Statistics provided include model equations and their corresponding coefficient of determination (r^2^) and correlation coefficients (r), as well as parameter significance (P-value; *if <0.05) and the percent sums of squares (%SS) associated with each parameter as a function of model term entry order.

### Objective 2: Remigration Interval Influence

**Figure 3:**
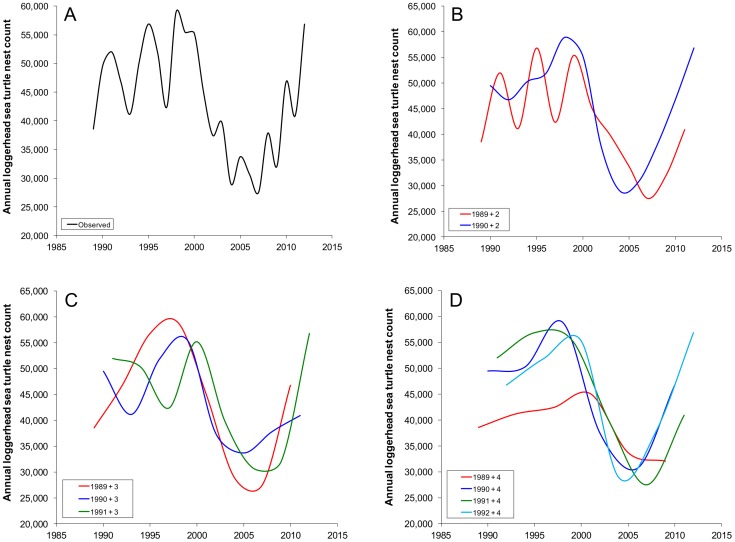
Remigration interval effects on loggerhead sea turtle (*Caretta caretta*) nest counts on Florida index beaches. Observed nest counts (A) were filtered into two (B), three (C), and four (D) year data series to evaluate remigration interval effects on interannual variability in nest counts and model performance. Data series orders for panels B–D were assigned as follows: first (red), second (dark blue), third (green), fourth (light blue). The multi-decadal shift between high (1998) and low (2007) annual nest counts was a common feature to the original and the filtered data series; however, inconsistencies in the interannual signal among the filtered data series within each remigration interval demonstrate the importance of contemporary environmental conditions on annual sea turtle nest counts.

The historical AMO 32 years prior to nest counts was only a significant model term for one data series for each of the three remigration intervals evaluated (Table 1, Figure 3). Equation term entry order resulted in high variability in the percent of sums of squares accounted for by the historical AMO (Table 1). With the exception of the four year remigration interval for the series beginning in 1991, for which the historical AMO accounted for 82–84% of the sums of squares and the greatest co-efficient of determination value was observed (adjusted r^2^ = 0.87), comparable percentages of sums of squares were accounted for by the historical AMO regardless of term significance (Table 1). The NAO in March of the year prior to nesting was never a significant model term for the filtered data set; however, for at least one series in each remigration interval, the NAO accounted for between 40–73% of the sums of squares (Table 1).

### Objective 3: Neophyte Importance and Nesting Projections

**Figure 4:**
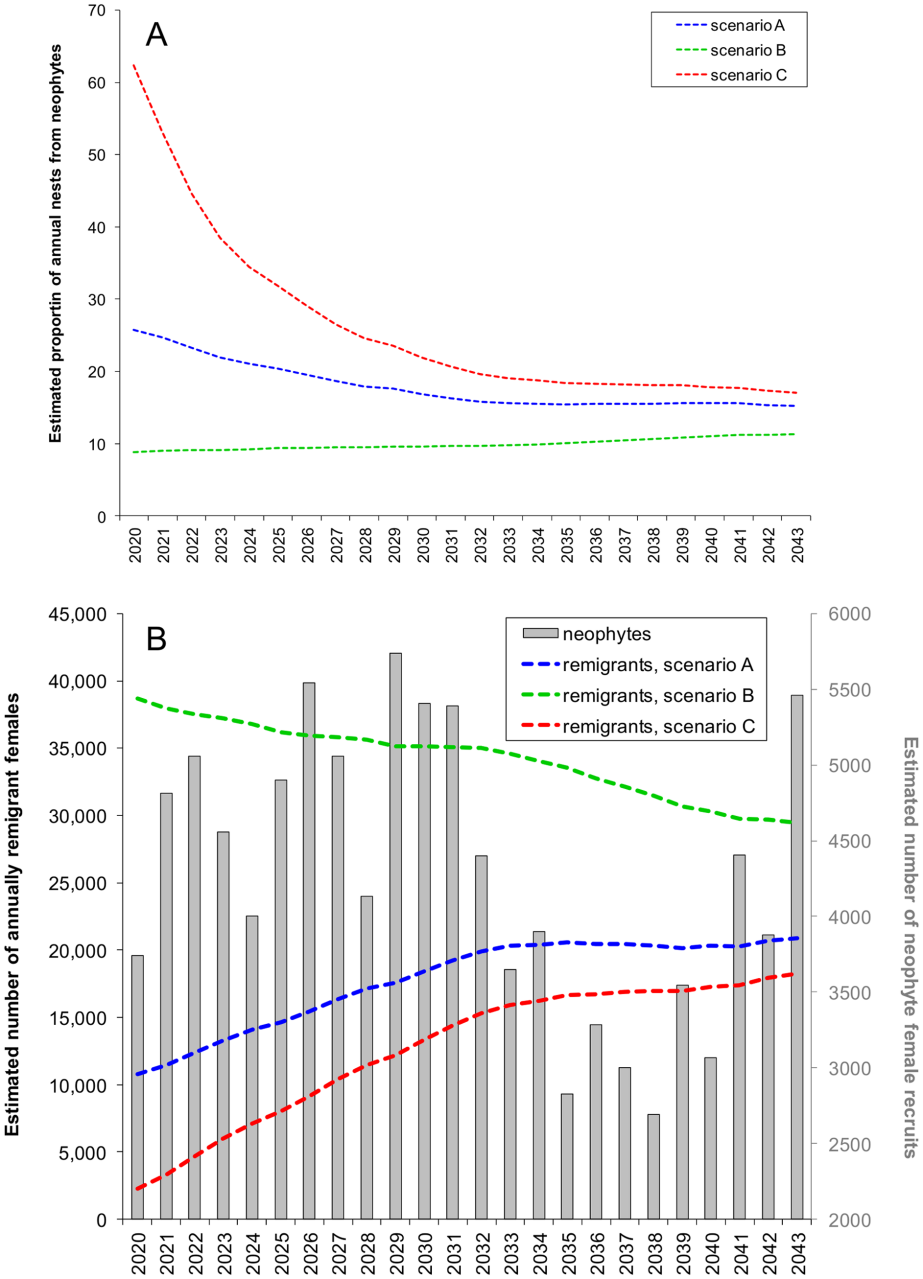
The relative importance of neophyte (A) and remigrant (B) nesters. Neophyte recruitment at 15 Florida index beaches during 2020–2043 was computed using observed nest counts at these beaches during 1989–2012 and assuming consistent stage- and age-based survival through age 31 across cohorts. Remigrant population size for the same period was evaluated under a range of scenarios contingent upon nesting in 2019. In scenario A (blue lines), nesting in 2019 was assumed to be the same as in 2012. In scenario B (green lines), nesting in 2019 was 258% greater than in 2012 following 20% annual increases after 2012. In scenario C (red lines), nesting in 2019 was 79% less than in 2012 following 20% annual decreases beginning in 2013.

Under the assumption of consistent stage-based and age-based survival across cohorts hatched on 15 Florida index beaches during 1998–2012, between 2,689 and 5,740 neophyte females were projected to recruit to the nesting population annually during 2020–2043 (Table S2). The greatest (62%, Figure 4a) projected proportion of annual nests associated with neophyte nesters occurred in 2020 under the assumption of a 79% decline in the remigrant population in 2019 (scenario C). Conversely, if nest counts in 2019 were unchanged from 2012 (scenario A) or 258% greater than 2012 (scenario B), neophyte nesters were only projected to account for 26% to 9% of nest counts in 2020, respectively. Systematic decline in the importance of neophyte nesters was projected through 2034 for scenarios A and B, at which point neophyte nester stabilized and accounted for 15–19% of annual nest counts (Figure 4a) concomitant with stable remigrant nesting by 15,000 to 20,000 females annually (Figure 4b). Because 5% annual loss of remigrant females under scenario B removed more females annually than were replaced by neophyte recruits, a slight but systematic increase in the importance of neophyte recruits was projected under scenario B (Figure 4a). Reduced neophyte recruitment during 2029–2038 in turn exacerbated the rate of decline in the remigrant population during the same period (Figure 4b).

**Figure 5:**
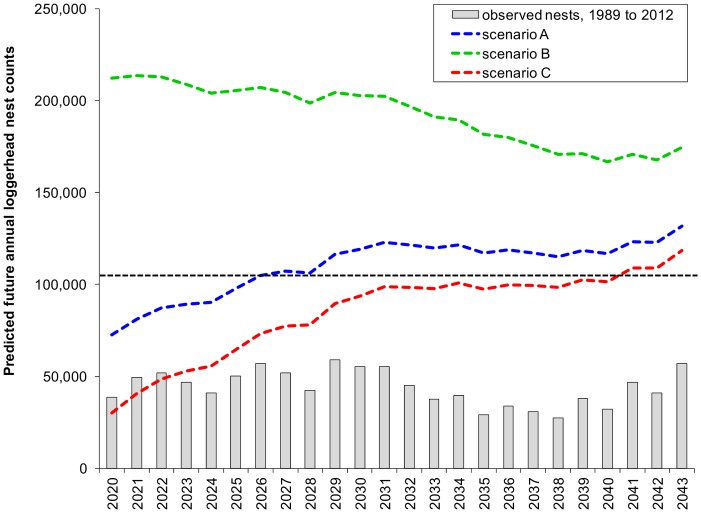
Projected (2020–2043) loggerhead (*Caretta caretta*) sea turtle nest counts on Florida index beaches. Age 31 neophyte nest counts during 2020–2043 were extrapolated from observed nest counts (grey bars) at 15 Florida index beaches during 1989–2012 under the assumption of consistent stage-based and age-based survival across all cohorts. Remigrant nest counts were evaluated for three scenarios in which nest counts in 2019 were unchanged from 2012 (scenario A, blue line), 258% greater than 2012 (scenario B, green line), and 79% less than 2012 (scenario C, red line). Under these three scenarios, which are also based on 5% annual population growth, projected annual nest counts for these 15 beaches would exceed the demographic recovery criteria for the entire Peninsula Recovery Unit of 106,100 nests per year (black line) by 2020, 2026, and 2041, respectively.

Annual nest counts projected for loggerhead sea turtles at these 15 Florida index beaches during 2020–2043 reflect the aforementioned temporal trends in remigrant population size (Figure 5). If nesting in 2019 was equal to that reported in 2012 (scenario A), annual nest counts were projected to steadily increase from 72,713 in 2020 to a high of 123,021 in 2031, decrease by 6.3% to 115,230 nests in 2038, then increase again to 131,711 nests in 2043 (Figure 5). If nesting in 2019 was 79% less than reported in 2012 (scenario C), annual nest counts were projected to steadily increase from 30,044 in 2020 to 98,751 in 2031, remain generally steady through 2038, and then systematically increase to 118,597 nests in 2043 (Figure 5). Only under the assumption of 258% greater nesting in 2019 relative to 2012 (scenario B) did annual nest counts systematically decline through 2043 (Figure 5), with the greatest decline (16%) noted between 2029 (204,342 nests) and 2038 (170,365 nests), corresponding to reduced recruitment from neophytes hatched between 1998 and 2007.

### Objective 4: Survival Matrix Evaluation

A maximum difference of 114% existed between loggerhead sea turtle nest counts recorded at 15 Florida index beaches between 1998 (58,880) and 2007 (27,513). However, if net survival for the 2007 cohort was 3.8% greater annually relative to the 1998 cohort during the next 30 years, it would be possible for these two cohorts to have equal abundance by age 31. Described by the age 0 vs. age 1 to 31 survival matrix (Table 2), high mortality experienced during three survival gauntlets (hatching emergence, crawl frenzy, aquatic hatchling stage) in the first year of life may be offset by improvements in annual survival during the next 30 years. For example, 95% cohort survival through the first year of life coupled with 80% annual survival for the next 30 years preserves the same proportion of a cohort at age 31 (0.11%) as would exist for a cohort that experienced 30% survival through the first year of life but 90% annual survival for the next 30 years (Table 2).

**Table 2:** An overview of cohort survival to age 31, whereby columns denote a range of survival probabilities during three gauntlets in the first year of life (i.e., hatchling emergence, hatchling frenzy, and survival at sea) and rows denote a range of annual at-sea survival probabilities during the next 30 years.

	Survival, age 0											
Survival, age 1 to 31	1%	10%	20%	30%	40%	50%	60%	70%	80%	90%	95%	99%
1%	0.00%	0.00%	0.00%	0.00%	0.00%	0.00%	0.00%	0.00%	0.00%	0.00%	0.00%	0.00%
10%	0.00%	0.00%	0.00%	0.00%	0.00%	0.00%	0.00%	0.00%	0.00%	0.00%	0.00%	0.00%
20%	0.00%	0.00%	0.00%	0.00%	0.00%	0.00%	0.00%	0.00%	0.00%	0.00%	0.00%	0.00%
30%	0.00%	0.00%	0.00%	0.00%	0.00%	0.00%	0.00%	0.00%	0.00%	0.00%	0.00%	0.00%
40%	0.00%	0.00%	0.00%	0.00%	0.00%	0.00%	0.00%	0.00%	0.00%	0.00%	0.00%	0.00%
50%	0.00%	0.00%	0.00%	0.00%	0.00%	0.00%	0.00%	0.00%	0.00%	0.00%	0.00%	0.00%
60%	0.00%	0.00%	0.00%	0.00%	0.00%	0.00%	0.00%	0.00%	0.00%	0.00%	0.00%	0.00%
70%	0.00%	0.00%	0.00%	0.00%	0.00%	0.00%	0.00%	0.00%	0.00%	0.00%	0.00%	0.00%
80%	0.00%	0.00%	0.00%	0.00%	0.01%	0.02%	0.03%	0.04%	0.06%	0.09%	0.11%	0.12%
90%	0.00%	0.00%	0.03%	0.11%	0.27%	0.53%	0.92%	1.45%	2.17%	3.09%	3.63%	4.11%
95%	0.00%	0.02%	0.17%	0.58%	1.37%	2.68%	4.64%	7.36%	10.99%	15.65%	18.40%	20.83%
99%	0.00%	0.07%	0.59%	2.00%	4.73%	9.25%	15.98%	25.37%	37.87%	53.92%	63.42%	71.77%

Cell values were computed as the column header to the third power multiplied by the row header to the 30^th^ power.

## Discussion

In the past decade, considerable emphasis has been placed on assessing relationships between climate and population regulation of long-lived sea turtle species. Most climate-related studies have focused on changes in sex ratios as they would be affected by incubation temperature [27]. The role of temperature on sea turtle nesting has also been investigated with respect to reconstruction of historical incubation temperatures [28] and the potential future variation in spatio-temporal nest distribution and/or hatchling fitness [12], [29], [30], [31], [32]. Associations between temperature and food availability for adult females have also been suggested to be linked to interannual variability in nesting activity for most sea turtle species [14], [15], [16], [17], [18], [19]. Alternatively, Van Houtan and Halley [7] suggested that most of the interannual variability in loggerhead sea turtle nest counts in Florida between 1989 and 2010 could be explained by climate forcing on hatchling survival. In the present study, we reached greatly different conclusions than those reached by Van Houtan and Halley [7], and herein highlight important analytical considerations that influenced the results of both studies.

Interannual variability in nest counts explained by the NAO index in March of the year prior to nesting was not statistically different from the variability explained by the winter and spring AMO 32 years earlier. This observation was not in agreement with the 31-year AMO lag influence reported by Van Houtan and Halley [7]. We attribute this disparity to the following factors: (1) in the present study the number of historical and contemporary lag terms was equal whereas the curvilinear model of Van Houtan and Halley [7] included twice as many historical as contemporary terms; (2) only significant model terms were included in our models; and (3) the contemporary term selected in the present study (i.e., March NAO) had a superior overall model fit than the contemporary term (i.e., December SST) used by Van Houtan and Halley [7]. Although the NAO was a significant model term overall, the relationship between NAO and annual nest counts was strongest prior to the 41% decline in annual nest counts which occurred between 1998 and 2007 [8]. Reduced performance of the March NAO after 2001 likely stemmed from reduced variability in nest counts concomitant with increased variability in the March NAO during 2004–2009. This assertion is supported by enhanced performance of the lagged AMO index during 2004–2009, when interannual variability in the lagged AMO was at its absolute minimum during the 24 years of data evaluated.

We hypothesize that improved performance of the lagged AMO metric vs. the contemporary NAO metric with respect to remigration intervals may also be attributed to data variance. Unlike the NAO, the AMO was a significant model term for at least one subset of data analyzed for each remigration interval, with the best fit observed for a four-year remigration interval series which began in 1991. However, closer inspection of this data set (Figure 2d) revealed inconsistencies in the relationship between variation in annual nest counts and variation in the lagged AMO. For example, between 1995 and 1999 the lagged AMO varied by 0.70, but corresponded to a less than 3% decline in annual nest counts. Between 1999 and 2002 the AMO index was predominantly associated with values approaching zero; however, nest counts declined by 32%. Conversely, the greatest interannual change (i.e., a decline of 2.62) in the AMO occurred between 2002 and 2003 when nest counts increased slightly (6%). Similarly, only a modest change (0.11) in the lagged AMO was observed between 2003 and 2007 when there was a 30% decline in annual nest counts. Lastly, between 2007 and 2011, annual nest counts increased by nearly as much as they decreased between 2003 and 2007, but the difference in change in the lagged AMO between these periods was ten-fold. In summary, despite the strong linear trend between the lagged AMO and annual nest counts for loggerhead sea turtles on 15 Florida index beaches, we suggest that this observation represents a spurious relationship due to the multi-decadal cycle of the AMO. This chance co-occurrence brought about a strong fit between extreme nest counts and AMO metrics, but otherwise resulted in inconsistent relationships.

Prior to 2001, observed nest counts were generally greater than nest counts predicted based on contemporary NAO, historical AMO, or both of these parameters; however, the opposite relationship persisted between 2001 and 2009, particularly for the NAO only model.

Reduced performance of all environmental indices models during the period of nest count decline suggests a lagged response to population perturbations which occurred decades prior. Although survival of all life history stages is important, our survival matrix demonstrated that survival at sea over 30 years has a greater cumulative influence on cohort abundance at age 31 than survival exclusively in the first year of life. This conclusion concurs with population modeling by Conant et al. [3] who also cautioned on the perils of accumulation of even small declines in survival across decades, as well as the need for high annual survival rates at sea to maintain positive population growth, as previously suggested by Leslie-Matrix models [4], [5].

When observed nest count data were partitioned into multiple data series at selected remigration intervals, all series exhibited evidence of stabilization (if not recovery) following a decline. Prior to the decline in annual nest counts during 1998–2007, one series (with different data) for both the two-year and four-year remigration intervals exhibited considerably less variability than the others. Although it is highly unlikely that all nesting sea turtles in a given year are on the exact remigration schedule [16], [18], this finding is intriguing given that prior to the decline, the NAO was a marginally non-significant term that accounted for one-third of annual nest count variation. We attribute the pre-decline nest count associations to have driven the significance of the NAO in the overall model, but also note that the NAO, the AMO, and the ENSO are all teleconnected climatically. Therefore, these climate teleconnections also explain the associations of all three climate indices with the annual nest counts reported in Figure 1.

Loggerhead sea turtles were added to the Endangered Species Act in 1978 (FWS and NMFS 1978, 43 FR 32800) and it is reasonable to presume that this listing was preceded by practices that negatively affected loggerhead survival for some time prior to species listing. Conservation efforts on Florida beaches began prior to the listing [8], but characterization of in-water threats and subsequent implementation of mitigation measures were not initiated until several decades later [5], [33], [34]. Therefore, given that the lowest annual nest count data for loggerhead sea turtles on Florida index beaches occurred 30 years after their receipt of federal protection, we cautiously suggest that the decline during 1998–2007 represents a lagged perturbation effect. This scenario has been demonstrated for leatherbacks nesting in Pacific Costa Rica where illegal, historical egg poaching, that was estimated to affect nearly 90% of all nests, had a lagged response on nesting numbers [11]. We further suggest that recent increases in Florida loggerhead annual nest counts since 2008 may reflect lagged protection benefits, especially given steady increases in catch rates for loggerhead sea turtles approaching maturity since 2000 [35] and that exhibit a strong genetic association with regional nesting assemblages [36].

Nesting projections for 2020 to 2043 under a range of nesting population scenarios also reinforce the suggestion that 1998–2007 decline in nesting resulted from differential survival and/or female production across cohorts several decades prior. Because these projections were based on the assumption of consistent survival rates and reproductive traits across cohorts, neophyte recruitment during 2020–2043 was projected to mirror nesting trends during 1989–2012; however, because neophytes were projected to generally account for a small portion of nesting during 2020–2043, the precipitous decline in nesting during 1998–2007 was not projected to recur 31 years later. Furthermore, our projections suggest that unless nesting declines appreciably between 2013 and 2019 relative to 2012 and if the population was growing in accordance with a 5% trajectory, annual nesting at the 15 index beaches we analyzed would attain the 106,100 annual nest count demographic recovery criteria for the entire Peninsular Recovery Unit of the Northwest Atlantic Distinct Population Segment [37] by 2027. Lastly, because we assumed a conservative 1∶1 female to male sex ratio versus the highly female-biased ratios reported for loggerhead sea turtles in the North Atlantic [38], [39], [40], there is an opportunity for our nest count projections to be underestimates.

On multiple foraging grounds throughout the southeast U.S., catch rates and size distributions have increased for a range of loggerhead sea turtle size classes during the past two decades [35], [40], [41], [42]. These changes were attributed to recruitment of strong year classes that successfully passed through the survival gauntlet; however, without an age structure for these sampled foraging grounds, support for increased recruitment remains weak. Age assessments are routinely conducted for dead stranded sea turtles because skeletochronology, the most promising technique demonstrated thus far, is an invasive procedure that requires a full cross section of the humerus [22]. Although this technique could be used to track the temporal prevalence of age classes, interpretation of trends presents a challenge given that a temporal decline in the occurrence of stranded age × turtles could at least in part be influenced by increased annual survival rates at sea. Size classes are often used as a proxy for age, but this practice is also not ideal because growth rates fluctuate spatially and temporally [43] as well as decline with age [44]. Because environmental conditions vary across decades, as substantiated by the indices examined in the present study, the development of reliable and minimally-invasive methods for conducting large-scale age assessments should be a high priority for future research and funding.

Satellite telemetry has revealed that loggerhead sea turtles captured at mating aggregations [45] and nesting assemblages [46], [47] in the Northwest Atlantic Ocean consist of both resident and migratory individuals. We hypothesize that individuals located further from breeding areas have greater migration energy requirements, and likewise suggest that resident individuals have shorter remigration intervals than their migrant counterparts. Hamann et al. [48] suggested that variable reproductive remigration intervals among individuals may reflect differences in the uptake of energy needed to reach a critical threshold body condition. Using stable isotopes, which offer a reliable and nominally-invasive means to distinguish between oceanic and neritic foraging grounds [49], [50], Hatase et al. [51] demonstrated longer remigration intervals for oceanic female loggerhead sea turtles than their neritic counterparts. Stable isotopes, in conjunction with satellite telemetry, have also been used to differentiate neritic foraging grounds across latitudinal gradients [47], [52]. On a limited basis, Pajuelo et al. [52] also reported interannual variability in the contribution of nesting females from various foraging grounds. We suggest that annual isotope characterizations, coupled with diet studies and somatic growth assessments, could also greatly improve capabilities for future nest count projections. As such, we encourage continued efforts to use stable isotopes to monitor interannual variability in the contributions of adult female sea turtles from various foraging grounds to annual nest counts. Bjorndal et al. [43] recently reported a temporal decline in growth rates for juvenile loggerheads corresponding to the period of reduced nesting activity in Florida [8], further reinforcing the potential link between energy uptake and subsequent annual nesting activity.

## Supporting Information

Figure S1 **Visualization of the disproportionate response of squaring (blue bars) negative vs. positive values for the normalized AMO index (red bars), and the subsequent temporal disparity for comparison with annual nest counts (black line) for loggerhead (** ***Caretta caretta*** **) sea turtles on 15 Florida index beaches between 1989 and 2012.**
(TIF)

Table S1 **Descriptive statistics (mean, SD, coefficient of variation (CV), minimum, and maximum) for the percent sums of square values associated with each model parameter examined in the four models considered by Van Houtan and Halley** [**7**] **.** Model parameters (AMO, NAO, ENSO) were evaluated at lagged intervals to quantify contemporary (<4 years before nest count) and cohort (>10 years after nest count) influences. Model coefficient of determination (adjusted r^2^) and significance (P-value) are also provided.
(TIF)

Table S2 **Estimated total and remigrant female loggerhead (** ***Caretta caretta*** **) sea turtle population sizes during 1989–2012 and projected (2020–2043) neophyte, total, and remigrant adult female population sizes under selected scenarios.** In scenario A, the remigrant population size was unchanged from 2012. In scenario B, the remigrant population size was 258% greater than in 2012 following 20% annual increases in nest counts during 2013–2019. In scenario C, the remigrant population size was 79% less than in 2012 following 20% annual decreases in nest counts during 2013–2019. For all three scenarios, the total adult female population in each year between 2020 and 2043 was computed as the compounded sum of a 5% annual decline in an initial (2019) remigrant pool plus a subsequent 5% annual decline in each neophyte cohort size between 2020 and 2043 after initial nesting. Given a three-year remigration interval, annual remigrants were computed as one-third of each annual adult female population size estimate between 2020 and 2043.
(TIF)
