# Supplementary figures and images for: Historical versus Contemporary Climate Forcing on the Annual Nesting Variability of Loggerhead Sea Turtles in the Northwest Atlantic Ocean
Source: PLoS One. 2013 Dec 5;8(12):e81097. doi: 10.1371/journal.pone.0081097 (PMC3855202; doi:10.1371/journal.pone.0081097)

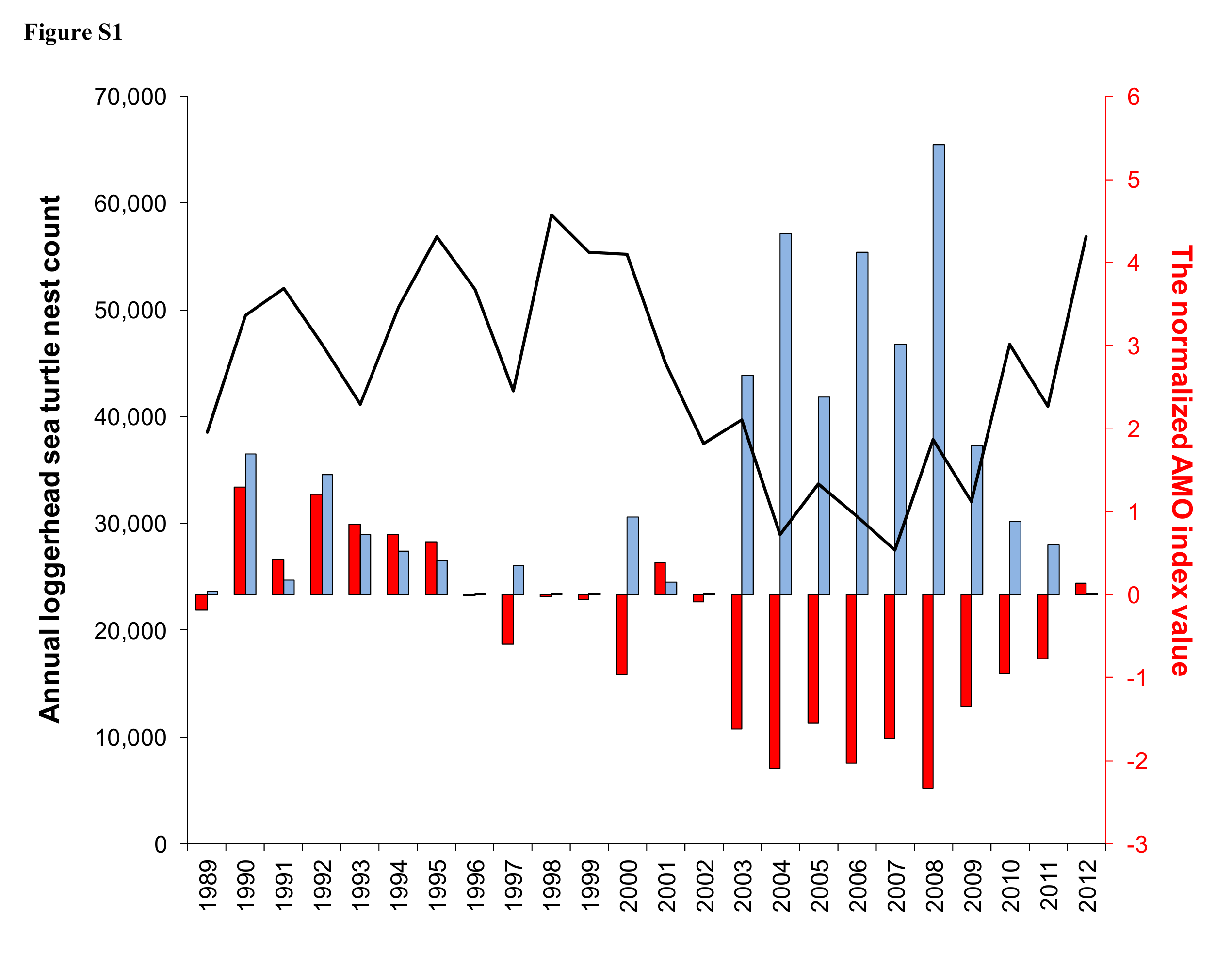

Supplement: Figure S1 — Visualization of the disproportionate response of squaring (blue bars) negative vs. positive values for the normalized AMO index (red bars), and the subsequent temporal disparity for comparison with annual nest counts (black line) for loggerhead ( Caretta caretta ) sea turtles on 15 Florida index beaches between 1989 and 2012. (TIF) [file pone.0081097.s001.tif]
